# The *hOCT1* and *ABCB1* polymorphisms do not influence the pharmacodynamics of nilotinib in chronic myeloid leukemia

**DOI:** 10.18632/oncotarget.21406

**Published:** 2017-09-30

**Authors:** Sara Galimberti, Cristina Bucelli, Elena Arrigoni, Claudia Baratè, Susanna Grassi, Federica Ricci, Francesca Guerrini, Elena Ciabatti, Carmen Fava, Antonio D’Avolio, Giulia Fontanelli, Giovanna Rege Cambrin, Alessandro Isidori, Federica Loscocco, Giovanni Caocci, Marianna Greco, Monica Bocchia, Lara Aprile, Antonella Gozzini, Barbara Scappini, Daniele Cattaneo, Anna Rita Scortechini, Giorgio La Nasa, Alberto Bosi, Pietro Leoni, Romano Danesi, Giuseppe Saglio, Giuseppe Visani, Agostino Cortelezzi, Mario Petrini, Alessandra Iurlo, Antonello Di Paolo

**Affiliations:** ^1^ Department of Clinical and Experimental Medicine, Section of Hematology, University of Pisa, Pisa, Italy; ^2^ Oncohematology Division, IRCCS Ca’ Granda, Maggiore Policlinico Hospital Foundation, University of Milan, Milano, Italy; ^3^ Department of Clinical and Experimental Medicine, Section of Pharmacology, University of Pisa, Pisa, Italy; ^4^ GeNOMEC, University of Siena, Siena, Italy; ^5^ Hematology Division, Ospedale Mauriziano, Torino, Italy; ^6^ Laboratory of Clinical Pharmacology and Pharmacogenetics, Department of Medical Sciences, University of Torino, Torino, Italy; ^7^ Department of Clinical and Biological Sciences, University of Torino, AOU San Luigi Gonzaga, Torino, Italy; ^8^ Hematology and Stem Cell Transplant Center, San Salvatore Hospital, Pesaro, Italy; ^9^ Department of Medical Sciences, University of Cagliari, Cagliari, Italy; ^10^ Division of Hematology, Ospedale Le Scotte, University of Siena, Siena, Italy; ^11^ Division of Hematology, AOU Careggi, University of Florence, Firenze, Italy; ^12^ Division of Hematology, Marche Polytechnic University, Ancona, Italy

**Keywords:** nilotinib, ABC transporters, ABCB1, hOCT1, early molecular response

## Abstract

First-line nilotinib in chronic myeloid leukemia is more effective than imatinib to achieve early and deep molecular responses, despite poor tolerability or failure observed in one-third of patients. The toxicity and efficacy of tyrosine kinase inhibitors might depend on the activity of transmembrane transporters. However, the impact of transporters genes polymorphisms in nilotinib setting is still debated. We investigated the possible correlation between single nucleotide polymorphisms of *hOCT1* (rs683369 [c.480C>G]) and *ABCB1* (rs1128503 [c.1236C>T], rs2032582 [c.2677G>T/A], rs1045642 [c.3435C>T]) and nilotinib efficacy and toxicity in a cohort of 78 patients affected by chronic myeloid leukemia in the context of current clinical practice. The early molecular response was achieved by 81% of patients while 64% of them attained deep molecular response (median time, 26 months). The 36-month event-free survival was 86%, whereas 58% of patients experienced toxicities. Interestingly, *hOCT1* and *ABCB1* polymorphisms alone or in combination did not influence event-free survival or the adverse events rate. Therefore, **i**n contrast to data obtained in patients treated with imatinib, *hOCT1* and *ABCB1* polymorphisms do not impact on nilotinib efficacy or toxicity. This could be relevant in the choice of the first-line therapy: patients with polymorphisms that negatively condition imatinib efficacy might thus receive nilotinib as first-line therapy.

## INTRODUCTION

After the introduction in the clinical practice of the tyrosine kinase inhibitors (TKIs), the outcome of patients affected by chronic myeloid leukemia (CML) has really improved, with 96% of them alive and free of progression at 3 years [1].

Nilotinib was initially approved as a second-generation TKI for the treatment of patients resistant to imatinib, and since 2007 also as a first-line option [2]. It is structurally similar to imatinib, binding to the inactive conformation of the protein encoded by the *BCR-ABL1* fusion gene, but it is also characterized by a higher efficacy than imatinib [3]. The prospective, randomized phase-3 trial ENESTnd comparing nilotinib 300 mg or 400 mg twice/day vs imatinib 400 mg daily clearly demonstrated the superiority of nilotinib in terms of complete cytogenetic response (CCyR) (by 12 months, 80% in the nilotinib vs 65% in the imatinib arm) and of major molecular response (MR3) (by 5 years, 77% for nilotinib vs 60.4% for imatinib, *p*<0.0001). Interestingly, progressions occurred in 5 out of the 563 patients in the nilotinib arms (0.88%) in comparison to 12 out of 283 cases receiving imatinib (4.2%) [4].

Overall, the choice of nilotinib as first-line treatment significantly increases the probability of reaching the desired surrogate end-points at the correct time-points, but also the probability of achieving a deep molecular response (DMR, by 5 years, 54% with nilotinib vs 31% for imatinib), which represents the basis for a further possible drug discontinuation. Nevertheless, about 40% of patients discontinue nilotinib due to suboptimal response or toxicity [4].

Different mechanisms have been recognized on the basis of TKI resistance, from the *ABL1* mutations [5] to the activation of several alternative pro-proliferative pathways (such as Wnt, PI3K, Aurora Kinase, STAT3) [6–8] or the inhibition of the correct immunological control [9]. In addition, the transmembrane transporters have been also indicated as responsible for the resistance to TKIs, with a relevant role recognized to the ATP binding cassette (ABC) efflux pumps and to the human organic cation transport member 1 (hOCT1) influx protein [10]. Indeed, the pharmacokinetics and pharmacodynamics of imatinib seem to be influenced by *ABCB1* [11–13], *hOCT1* [14–16], and *ABCG2* polymorphisms [17]. Moreover, an *in vitro* study conducted on resistant K562 cells showed that *ABCG2* over-expression was associated with a reduced efficacy of imatinib, nilotinib, dasatinib, and bosutinib [18]. On the contrary, other studies demonstrated that dasatinib is a substrate of *ABCB1* [19], while the binding affinity of this transmembrane transporter seems to be lower for nilotinib [20, 21], bosutinib [22], and ponatinib [21]. Moreover, nilotinib appeared as one of the most potent modulators of ABCB1 and ABCG2 in respect of imatinib, dasatinib, erlotinib, lapatinib, and sunitinib in a murine model of chemoresistance [23].

Finally, a recent meta-analysis clearly demonstrated that the highest values of minimum plasma concentrations of imatinib were significantly correlated with the complete cytogenetic response, but not with the achievement of the complete molecular response [13]. About polymorphisms, authors concluded that the c.421A *ABCG2* variant allele was significantly associated with higher rate of MR3, whereas *ABCB1* variant alleles did not, with the exception of c.2677T/A alleles [13]. However, the complexity of the methods used for data analyses and the different non-comparable models have frequently led to conflicting results [24, 25].

With these premises, we decided to address the influence of *ABCB1* and *hOCT1* polymorphisms on the response rate and toxicity of first-line nilotinib in the context of a multicenter “real-life” series of 78 CML cases. Of note, patients were enrolled in a prospective and retrospective way in order to perform the analyses in a powered study.

## RESULTS

### Nilotinib efficacy: hematological, cytogenetic, molecular responses, and EFS

Seventy-eight CML patients, all in chronic phase, median age 47 years (range, 18-79) were enrolled in the study. Forty-six (59%) were males, and 32 (41%) were females. According to the Sokal, Hasford, and European Treatment and Outcome Study (EUTOS) scores, nearly half of the patients were classified into the low-risk groups. The median follow-up time in our study was 43 months; 2/3 of the enrolled cases were followed for 36 months, 74% for 24 months, 80% for 18 months. All patients had a minimum follow-up of 12 months. No significant differences in clinical characteristics have been observed between the prospective and retrospective cohorts (Table 1). The response rates have been detailed in Table 2.

**Table 1:** Clinical characteristics of the 78 enrolled patients

Clinical feature	Overall series n. (%)	Prospective cohort n. (%)	Retrospective cohort n. (%)	Statistical significance^*^
**Patients**	78	29	49	
**Age** (years)^**^	47 (18-79)	49 (20-68)	46 (18-79)	p=0.460
**Sex**				p=0.351
M	46 (59%)	15 (52%)	31 (63%)	
F	32 (41%)	14 (48%)	18 (27%)	
**Sokal risk score**				p=0.542
Low	34 (44%)	11 (38%)	23 (47%)	
Intermediate	29 (37%)	12 (42%)	17 (34%)	
High	15 (19%)	6 (20%)	9 (19%)	
**Eutos risk score**				p=0.301
Low	40 (51%)	11 (38%)	29 (59%)	
High	27 (35%)	11 (38%)	16 (33%)	
N/A	11 (14%)	7 (24%)	4 (8%)	
**Hasford risk score**				p=0.054
Low	40 (51%)	11 (38%)	29 (59%)	
Intermediate	26 (34%)	14 (48%)	12 (25%)	
High	12 (15%)	4 (14%)	8 (16%)	

**Note:** ^*^, Students’ *t* test; ^**^, values are expressed as median (min-max range)

**Table 2:** Hematological, cytogenetic, and molecular responses at different time-points

Responses	Overall Series n. (%)	Prospective cohort n. (%)	Retrospective cohort n. (%)	Statistical significance
**CHR by 3 months**				
**Yes**	76 (97%)	29 (100%)	47 (96%)	p=0.392
**No**	2 (3%)	0	2 (4%)	
**CCyR by 6 months**				
**Yes**	66 (85%)	25 (86%)	41 (84%)	p=0.376
**No**	12 (15%)	4 (14%)	8 (16%)	
**CCyR by 12 months**				
**Yes**	69 (88.5)	25 (86%)	44 (90%)	p=0.593
**No**	9 (11.5)	4 (14%)	5 (10%)	
**EMR**				
**Yes**	63 (81%)	28 (96%)	35 (71%)	**p=0.012**
**No**	15 (19%)	1 (4%)	14 (29%)	
**MR3 by 12 months**				
**Yes**	60 (77%)	22 (76%)	38 (78%)	p=0.537
**No**	18 (23%)	7 (24%)	11 (22%)	
**MR3 (at any time)**				
**Yes**	68 (88%)	23 (79%)	45 (92%)	p=0.182
**No**	10 (12%)	6 (21%)	4 (8%)	
**DMR (at any time)**				
**Yes**	50 (64%)	13 (45%)	37 (76%)	**p=0.002**
**No**	28 (36%)	16 (55%)	12 (24%)	

**Abbreviations:** CHR, complete hematological response; CCyR, complete cytogenetic response; EMR, early molecular response; MR3, complete hematological response; DMR, deep molecular response

#### Hematological response

Seventy-six out of the 78 patients enrolled into the study (97.4%) achieved a complete hematological response (CHR) by 3 months of treatment; the remaining two subjects reached a complete response by the sixth month of therapy. Overall, the CHR rate in our series was 100%.

#### Cytogenetic response

Sixty-nine patients (88.5%) achieved a complete cytogenetic response (CCyR) by 12 months, with 66 cases (84.6%) reaching a CCyR already by the sixth month [the time point identified as “optimal” by the European Leukemia Net (ELN) guidelines] [26]. The median time to achieve a CCyR was 3.2 months (range, 1.8-14.4); interestingly, by the 18^th^ month of treatment, the Philadelphia chromosome disappeared in 77 out of 78 patients. With a median follow-up of 43 months (range 4-106), no cases of loss of CCyR have been reported. The CCyR achievement was not significantly conditioned by sex, age, or Sokal and EUTOS score; nevertheless, the high Hasford score was associated with a lower probability of reaching the CCyR by the 12^th^ month with respect to the other groups (11% vs 43%; p=0.01). No significant differences in the quality of cytogenetic response were observed between the prospective and retrospective cohort.

#### Molecular responses

overall, the early molecular response (EMR) was achieved by 63 patients (81%); then, 68 subjects (87%) achieved an MR3 during the treatment. Of note, 60 patients reached an MR3 by 12 months, which identifies the optimal response. Furthermore, patients who experienced EMR more frequently achieved an MR3 by the 12^th^ month (83.6% vs 38.5%; p=0.002). The median time to achieve an MR3 was 6.1 months (range, 2.6 - 83); this interval was 7 months longer for cases without EMR. Seven patients (9%) lost the MR3 during treatment, with a median time of 31.2 months. As reported in Table 2, the EMR rate was higher in the prospective than in the retrospective cohort (p=0.012).

The 64.1% of patients receiving nilotinib achieved a DMR (≥MR4), with a median time to DMR attainment of 26 months: in particular, 24.3%, 28.2% and 11.5% of patients achieved an MR4, MR4.5 and MR5, respectively. As above reported for the MR3, also the DMR was more frequently achieved by subjects in EMR (71.8% vs 35.8%; p=0.04). As reported in Table 2, the DMR rate was higher in the retrospective than in the prospective cohort (p=0.002). The molecular response rates were not significantly conditioned by age, sex or CML risk scores.

#### EFS

**Figure 1:**
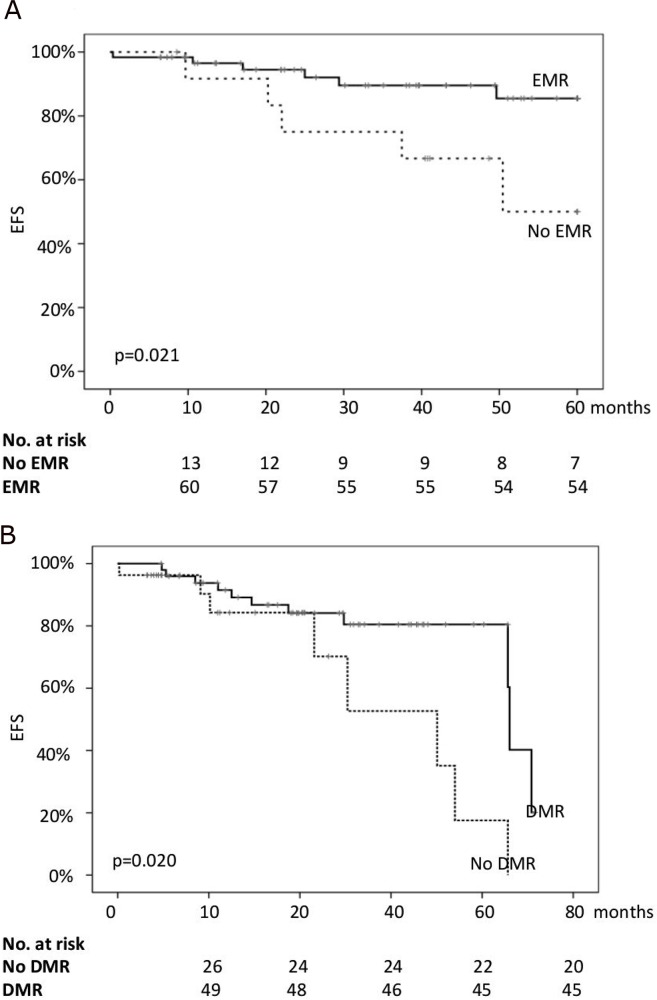
The probability of event-free survival (EFS) of the whole series according to the achievement of early molecular response (EMR, **(A)**) and deep molecular response (DMR, **(B)**).

All patients were still alive in July 2016. Thus, only the event-free survival (EFS) was considered for the statistical analysis. The 24-months EFS for the whole series was 89%, while the EFS at 36 and 48 months was 86% and 81%, respectively. Age >60 years, sex, and CML risk scores did not significantly impact on EFS. Similarly, the achievement of CCyR (either by 6 or by 12 months) did not significantly impact on EFS. On the contrary, the probability of proceeding without events was significantly higher for patients in EMR (94.5% vs 74.5% at 24 months; 86.2% vs 67.8% at 48 months; p=0.021) and for those who achieved a DMR during treatment (91% vs 84% at 24 months; 84% vs 70% at 48 months; p=0.020) (Figure 1). It is worth noting that the two groups of patients differed for EMR and DMR rates (Table 1) but not in terms of EUTOS and Sokal scores (Table 2). Therefore, it is plausible that the observed differences between groups could be mainly related to the limited number of patients enrolled.

Moreover, because in these cohorts only one event/each was censored, we analyzed the EFS in the whole series. When EMR and DMR were considered in the multivariate analysis, both lost the respective statistical significance. No differences in EFS according to the time to MR3 or DMR were observed.

### Nilotinib toxicity

Overall, 45 patients (57.7%) developed toxicities: in particular, 10 (22.2%) suffered from hematological and 35 (77.8%) from extra-hematological adverse events (AEs); 5 cases reported both hematological and extra-hematological toxicities. AEs are listed in Table 3.

**Table 3:** Adverse reactions associated with nilotinib administration (graded according to CTC-AE grading system) and time intervals to events

TOXICITY	n (%)
**Every toxicity**	
Yes	45 (58%)
No	33 (42%)
**Hematological toxicity**	10 (13%)
Grade 1-2	10 (100%)
Grade 3	0
Grade 4	0
**Type of hematological toxicity**	
Trombocytopenia	2
Neutropenia	2
Anemia	6
Time to hematological toxicity (months)^*^	1.3 (1-12)
**Extra-hematological toxicity**	35 (45%)
Grade 1-2	17 (48%)
Grade 3	16 (46%)
Grade 4	2 (6%)
**Type of extra-hematological toxicity**	
Skin toxicity (rash, dry skin, erythema – grade 3, 3 pts)	8
Increase in transaminases (grade 3 in 5 pts), bilirubin (grade 3 in 2 pt)	8
Increase in amylase/lipase (grade 3 in 4 pts)	8
Increase in serum glucose/cholesterol	3
Ocular toxicity (hemorrhage, dry eye, conjunctivitis – grade 3 in 2 pts)	3
Cardio-vascular toxicity (PAOD – grade 4, hypertension)	3
CNS toxicity (migrain, stroke – grade 4)	2
**Time to extra-hematological toxicity** (months)^*^	7.5 (0.2-85)
**Dose reduction**	
Yes	39 (50%)
No	39 (50%)
**Time to dose reduction** (months)^*^	12 (0.2-63)
**Nilotinib definitive discontinuation**	
Yes	18 (23%)
No	60 (77%)

Notes: ^*^, values are expressed as median (min-max range)

Among the extra-hematological toxicities, 30% interested the skin (more frequently rashes and dry skin), and 25% pancreas and liver (amylase, lipase, bilirubin and transaminases increased values). Other recorded AEs were metabolic (17.5%) (hyperglycemia, hypercholesterolemia), vascular (8%) (two cases of peripheral arterial occlusive disease, one of myocardial ischemia, two case of hypertension, one of arrhythmia), neurological (10%) (headache), and ocular (5%) (conjunctivitis) events.

In all cases the hematological toxicities were of grade 1 and 2; the extra-hematological adverse events were of grade 1 and 2 in the 48.6%. Only in 2 cases toxicity was of grade 4 (one patient with hepatic toxicity, another one with cutaneous toxicity); in the remaining 16 cases (45.7%), the AEs were of grade 3.

All cases of hematological toxicity occurred in the first year of therapy, with a median time of 1.3 months (range, 1-12); similarly, 80% of the extra-hematological AEs presented in the first year of treatment, but with a median time of 7.5 months (range, 0.2-85).

It is worth noting that the occurrence of AEs had a significant impact on the drug administration: half of the patients reduced the dose at least once during the follow-up, and the median time to dose reduction was 12 months (range, 0.2-63).

Finally, 18 patients (23%) permanently discontinued treatment: of these, 10 for toxicity, 5 for treatment failure, 2 because of their accrual into suspension trials, and one for personal decision. The median time to discontinuation was 43.5 months (range, 10.6 - 90.8).

### Transporters’ polymorphisms and their clinical significance

Because genetic polymorphisms of *hOCT1* and *ABCB1* are associated with variable influx/efflux activity of transporters, the following polymorphisms were evaluated by quantitative PCR: c.480C>G [*hOCT1*]; c.1236C>T, c.2677G>T and c.3435C>T [*ABCB1*]. In particular, minor allele frequencies (MAFs) for the investigated polymorphisms were comprised in the range 0.207-0.500, and the Hardy-Weinberg equilibrium (HWE) was demonstrated for all single nucleotide polymorphisms (SNPs; Table 4).

**Table 4:** Distribution of allele frequencies for investigated polymorphic loci in *ABCB1* and *hOCT1* genes. For c.2677 locus the T/A alleles are indicated as W

Gene and SNP	Genotype	Frequency %	Allele	Frequency %	HWE
**ABCB1 c.1236C>T**	CC	36.0	C T	59.3 40.7	χ^2^: 0.082 p=0.775
	CT	46.7			
	TT	17.3			
**ABCB1 c.2677G>W**	GG	40.5	G T	64.3 35.7	χ^2^: 0.058 p=0.810
	GW	47.6			
	WW	11.9			
**ABCB1 c.3435C>T**	CC	24.0	C T	50.0 50.0	χ^2^: 0.120 p=0.729
	CT	52.0			
	TT	24.0			
**hOCT1 c.480C>G**	CC	64.0	C G	79.3 20.7	χ^2^: 0.315 p=0.575
	CG	30.7			
	GG	5.3			

The *ABCB1* loci were in linkage disequilibrium (range D’=0.486-0.944), and frequencies of the corresponding haplotypes are reported in Table 5. Interestingly, only 14.2% of patients were homozygous for the wild-type haplotype (haplotype 1), while 33.3% were homozygous for the polymorphic haplotypes (haplotypes 2-7).

**Table 5:** Distribution of *ABCB1* haplotypes (c.1236-c.2677-c.3435) inferred from genotypes of the enrolled patients. For c.2677 locus the T/A alleles are indicated as W

[Hapl. ID]	Haplotype definition	Observed frequency
**1**	**CGC**	0.383
**[2]**	**CGT**	0.224
**[3]**	**TWT**	0.286
**[4]**	**TGT**	0.014
**[5]**	**TGC**	0.023
**[6]**	**TWC**	0.059
**[7]**	**CWC**	0.012

The *ABCB1* [c.1236C>T] did not influence the probability of achieving CCyR, EMR, MR3, or DMR or the time to CCyR, MR3, or DMR. The same lack of significance on magnitude or time to responses was found for *ABCB1* [c.2677G>T] polymorphism.

On the contrary, the DMR rate was significantly higher for polymorphic c.3435CT/TT patients than for the wild-type c.3435CC ones (77.8% vs 43.0%; p=0.02).

Although single *ABCB1* polymorphisms returned different results when compared with clinical endpoints, *ABCB1* haplotypes were investigated for possible associations with nilotinib efficacy and/or tolerability. The comparisons were made between wild-type (i.e., CC/GG/CC) and polymorphic haplotypes (any of the other combinations that included polymorphic alleles). In agreement with the results presented above, *ABCB1* haplotypes did not significantly condition either the CCyR and MR rate or the time to achieve MR3 or DMR.

Similar findings were obtained for *hOCT1*, whose genotype at locus c.480 did not exert any influence on CCyR, EMR, MR3 or DMR achievement, or on the time to achieve MR3 or DMR.

**Figure 2:**
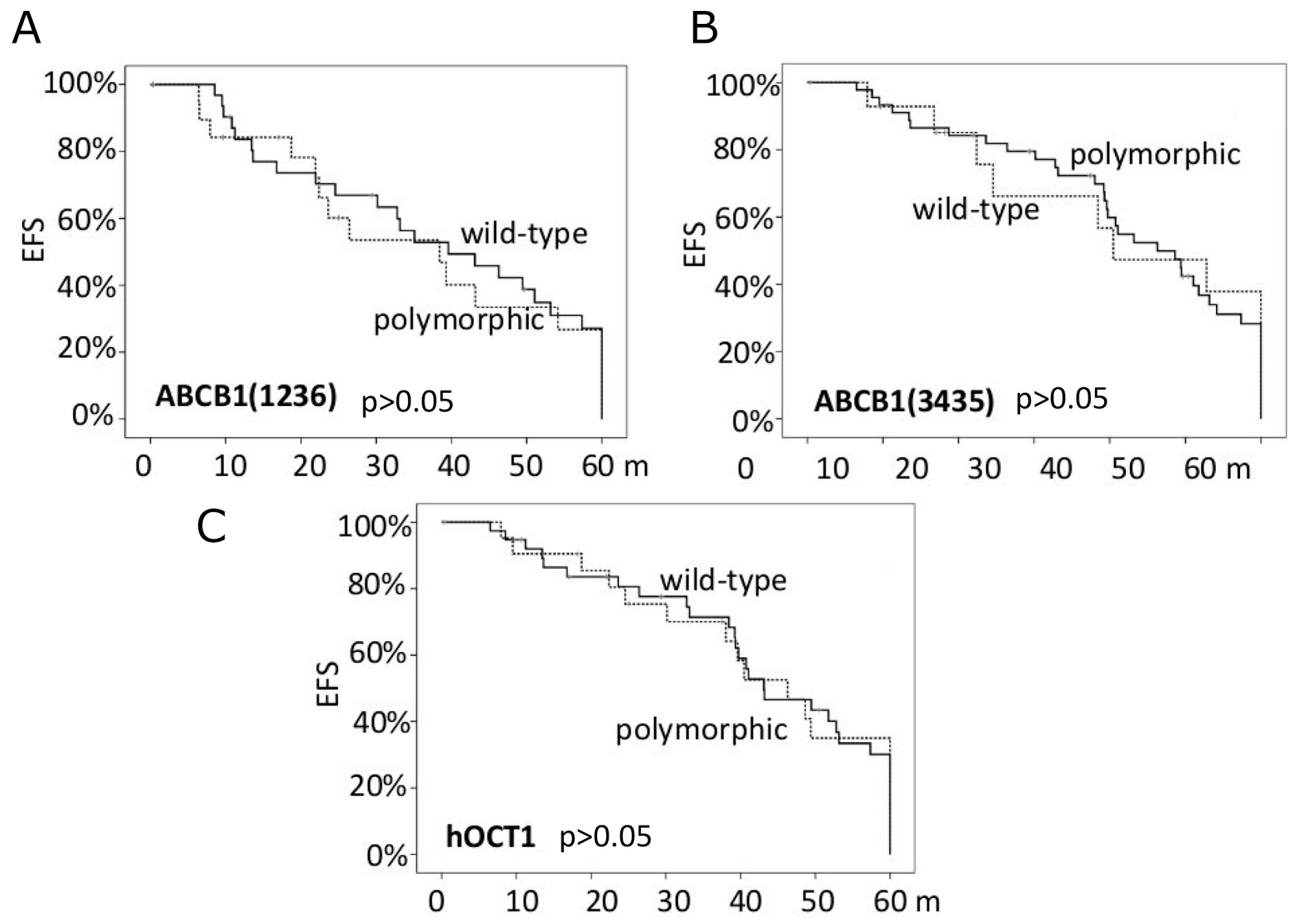
The probability of event-free survival (EFS) according to the ABCB1 polymorphisms (panels **A** and **B**) and the hOCT1 polymorphism (panel **C**).

Finally, the *ABCB1* polymorphisms did not significantly condition the EFS (Figure 2), the presence of toxicities nor their grade, either when analyzed alone or as haplotypes. The same findings were obtained for *hOCT1* genotypes with respect to the c.480C>G polymorphism.

## DISCUSSION

The present study suggests that *ABCB1* and *hOCT1* transmembrane transporters have not a significant impact on the efficacy and tolerability of nilotinib when used as a first treatment for CML patients. Indeed, our findings clearly show that polymorphisms associated with an altered activity of these transporters do not predict either the clinical outcome or the tolerability. Consequently, we believe that these results could have a clinical output, supporting the use of nilotinib regardless the genetic status of patients with respect of the two considered transporters.

Although the advent of imatinib has dramatically improved the prognosis of CML, about 30% of patients suspend the therapy for poor efficacy or adverse events [27]. More recently, second-generation (dasatinib, nilotinib, bosutinib) and third-generation TKIs (ponatinib) partially overcame those issues, making possible the successful treatment of resistant/intolerant patients, too [28, 29]. The choice of the TKI (imatinib, dasatinib or nilotinib) as a first-line therapy is sometimes difficult. Generally, the decision is led by the risk score assessment (second-generation TKIs for higher risk scores), age (imatinib for older people), co-morbidities (imatinib or dasatinib for patients with previous cardio-vascular events, nilotinib for those with lung diseases or pulmonary hypertension), but no clear suggestions came from the available international guidelines. Therefore, a careful choice of the best TKI *ab initio* is crucial to reach optimal responses and to reduce the occurrence of adverse events, hence promoting patient's adherence to treatment.

Several attempts to personalize treatment have been made evaluating the predictive role of the polymorphisms in genes coding for transmembrane transporters. If the picture is clear for imatinib, which is a substrate of both influx (hOCT1) and efflux (ABCB1) transporters, many doubts and contrasting findings characterize second- and third-generation TKIs. For example, the efflux of nilotinib, dasatinib and ponatinib was not significantly influenced by the expression of wild-type or polymorphic *ABCB1* in HEK293 and K562 cell lines, differently from imatinib [24]. On the contrary, other authors demonstrated that imatinib, dasatinib, nilotinib were all substrates of ABCB1 in the same K562 cells [25]. Because *ABCB1* over-expression significantly reduced when cells were continuously exposed to nilotinib, the authors hypothesized that the entity of the transporter gene expression would be important in the early stages of resistance to TKIs only [30]. Moving from the *in vitro* models to the clinical setting, other authors showed that the *ABCB1* wild-type haplotypes (at positions c.1236, c.2677 and c.3435) were associated with a significantly lower MR3 rate (50% vs 93%) under treatment with TKIs. In that small series, only 6 patients received nilotinib, 2 subjects carrying the wild-type and 4 the heterozygous polymorphic haplotype. In contrast with patients receiving imatinib, all cases treated with nilotinib achieved the MR3, regardless their haplotype [31]. The samples size of that study with respect to nilotinib was very limited, but our study showed the same results in a larger group of patients: the wild-type or polymorphic haplotypes did not impact either on the quality of response (CCyR, EMR, MR3, DMR) or on the EFS.

In agreement with what observed for *ABCB1*, we did not find any clinical impact for the *hOCT1* polymorphism on nilotinib efficacy and tolerability; this result was already expected, because it is well known that nilotinib is more lipophilic than imatinib and *in vitro* experiments suggested that it is not substrate for the *hOCT1* influx transporter [32]. Other studies investigated the substrate affinity of nilotinib for other transmembrane transporters, but conclusive results are not yet achieved [33]. The lack of any significant effect of *hOCT1* and *ABCB1* polymorphisms on the achievement of cytogenetic and molecular responses or on the EFS could be particularly relevant in the clinical practice. Indeed, if a patient carries the *hOCT1* polymorphic allele, for example, we can suppose that he will have a higher probability of failure and higher toxicity when exposed to imatinib [17]. In this case, a second-generation TKI, such as nilotinib, might represent a valid option for optimizing the efficacy and reducing the occurrence of AEs.

On the other hand, when patients receive imatinib, it has been reported a higher molecular response rate in the cohort of cases with polymorphic *ABCB1* [34]; in our series, *ABCB1* polymorphisms did not condition the time to response, its magnitude or the EFS, thus supporting the use of nilotinib independently from the genetic variants of the membrane transporters.

Moreover, our series of patients is really representative of the real clinical practice as witnessed by the superimposable rates of cytogenetic and molecular responses between our study and the ENESTnd trial. Indeed, in that international trial, the CCyR rate was 80%, and 77.2% of patients achieved an MR3 [4]. In line with those results, the CCyR rate was 88% in our study, and 77% of patients achieved an MR3. Furthermore, the 5-year EFS in our series was lower than that observed in the ENESTnd trial (81% vs 95%, respectively). That difference could depend on the wider definition of EFS in our study, because we considered the loss of MR3 and nilotinib discontinuation for any cause. Indeed, when the same ENESTnd criteria were applied to our series, the 5-year EFS would increase to 94.5%, the same EFS observed in the ENESTnd trial.

Finally, the overall AEs in our study were less frequent than in the ENESTnd trial (41% vs 60%); we can suppose that this discrepancy could depend on the prolonged experience in the drug management in our series in respect of the pivotal trial. Nevertheless, the occurrence of the cardiovascular events was similar (8% in the ENESTnd vs 7.5% in our series). Interestingly, in another Italian multicenter “real-life” study on 110 patients, 27% of them experienced cardiovascular events when nilotinib was administered as second-line treatment [35]. That comparison further strengthens the better tolerability of nilotinib as upfront therapy, despite our study recorded a slight higher discontinuation rate with respect to that reported in the ENESTnd trial (3.8% vs 2.5%, respectively).

It is worth to note that the demonstrated lack of any significant association between gene polymorphisms of transmembrane transporters and clinical outcome has been found in a homogenous cohort of patients: the fact that all cases received nilotinib as first-line treatment eliminates the possible “contaminating” effects induced by previously used TKIs. Nevertheless, some characteristics of our study have to be considered: first of all, clinical records were available for all patients and they gave the possibility to exclude the risk of drug-drug interactions, as well as smoke, alcohol consumption and comorbidities. On the contrary, the influence of dietary and herbal factors could not be ruled out in a complete manner. Moreover, other patients’ characteristics, such as physiological (body mass index, renal and liver function), pathological (comorbidities), genetic (gene polymorphisms, mutations) and epigenetic factors (changes in gene expression) may significantly have an influence on the clinical outcome during nilotinib administration [36]. Although some of those factors have been ruled out thanks to our clinical records, other ones remain unexplored and their future evaluation will require a wider enrollment, further efforts, and probably different analytical platforms, as well as genome sequencing (i.e., next generation sequencing or whole genome association studies). Moreover, our database included patients enrolled in retrospective and prospective cohorts, but, as stated above, the strict adoption of the 2013 ELN guidelines for all patients minimized the risk of potential biases. Finally, the present clinical findings are similar or superimposable with those reported in the ENESTnd trial as stated above, thus strengthening the reliability of our spontaneous and cooperative study.

In conclusion, for the first time our study supports the use of nilotinib as first-line treatment in a homogeneous series of CML patients irrespective to the *hOCT1* and *ABCB1* genotypes, replicating data obtained in the largest, company-driven ENESTnd trial. In contrast to these findings, our group recently published the significant influence of ABCB1/hOCT1 combined genotype on imatinib efficacy and tolerability [37]. Therefore, a simple and cheap test, such as a quantitative PCR, would aid the physician to correctly choose the right TKI for each patient, even if the test could not be available for all patients. Indeed, when a reduced cellular uptake through hOCT1 and higher efflux by ABCB1 do increase the risk of imatinib failure, nilotinib may represent the optimal choice.

## MATERIALS AND METHODS

The current study is part of a spontaneous, not sponsored sub-study of the “TIKLET” trial (Tyrosine Kinase Inhibitors in chronic myeloid leukemia: Efficacy and Tolerability; ClinicalTrials.gov identifier: NCT01860456), which has been carried out on 78 adult subjects affected by CML in chronic phase receiving nilotinib 600 mg as first-line treatment, outside clinical trials, in 8 Italian Hematology Divisions. Of note, none of the enrolled patients took part in previous clinical trials.

From January 2013 to July 2015, 29 patients were prospectively enrolled in the study. Sample size analysis demonstrated the study was downsized, hence further 49 subjects receiving first-line nilotinib after 2008 were also considered if a) they received nilotinib as first-line treatment and b) the standardized *BCR-ABL1/ABL1* quantitative PCR was available, according to the Labnet Italian Network standardized operative procedures. Those criteria allowed the complete revision of patients’ clinical history and laboratory results (see following paragraphs), hence statistical analyses were performed in a homogeneous population with respect to clinical and molecular endpoints. Statistical analyses between groups were performed only to exclude severe differences.

The study protocol has been approved by the Ethics Committee of the Azienda Ospedaliero Universitaria Pisana (protocol n. 46013/2011) and by local Ethics Committees of participating centres. Patients gave their informed consent to study participation before their enrollment.

The following inclusion criteria were adopted: (a) patients of both gender, (b) aged ≥18 years, (c) treated with nilotinib as first-line treatment, (d) with at least 3 months of follow-up, (e) the availability of standardized *BCR-ABL1/ABL1* quantitative PCR results both at diagnosis and during the follow-up; (f) complete clinical history of patients.

All patients were treated with an initial daily dose of 600 mg.

Age, sex, Sokal, Hasford and EUTOS risk scores were recorded; for each patient, two independent hematologists scored the hematological, cytogenetic and molecular responses defined according to the ELN recommendations edited in 2013 [26]. In this manner, every bias in scoring the clinical efficacy of nilotinib was kept at a minimum (see below).

The AEs data collection has been done according to the Common Toxicity Criteria –Adverse Events (CTC-AE) version 4.03. In agreement with the efficacy data, the database of toxicities was deeply reviewed by two hematologists according to the above-cited CTC-AE criteria.

### *BCR-ABL1/ABL1* response monitoring

Hematological, cytogenetic, and molecular responses were assessed and classified according to the ELN guidelines [26]. Furthermore, molecular monitoring was carried out on peripheral blood samples and performed by quantitative PCR, as established by the European guidelines [38] applied by all Italian laboratories afferent to the LABNET network, and results expressed in accordance with the International Scale (IS). In particular, molecular responses were “graded” according to the logarithmic reduction with respect to a basal level (considered as 100%). Thus, the molecular responses MR3, MR4 and MR5 imply 3-log (0.1% IS), 4-log (0.01% IS) and 5–log (0.001% IS) reduction, respectively. The responses with a ≥4-log decrease identified the DMR, the sensitivity of assays depending on the number of the *ABL1* copies in each amplification well (always >10.000). Finally, the EMR was defined as a *BCR-ABL1/ABL1* %IS ≤10% at 3 months from the treatment beginning.

### Pharmacogenetic analysis

The genetic variables considered were represented by the following polymorphisms: a) *hOCT1* rs683369 [c.480C>G]; b) *ABCB1* rs1128503 [c.1236C>T] (exon 12); c) *ABCB1* rs2032582 [c.2677G>T/A] (exon 21); d) *ABCB1* rs1045642 [c.3435C>T] (exon 26).

Genomic DNA extracted from peripheral blood (QIAamp DNA Blood Mini Kit, Qiagen, Milan, Italy) was used to obtain the genotype of each patient by using specific TaqMan assays (Life Sciences, Milan, Italy) on an ABI Prism 7900HT Sequence Detection System (Life Sciences).

MAF calculation, HWE evaluation and haplotype analyses were performed by using Arlequin software [39]. The recessive model was adopted, grouping patients according to the absence (wild-type) or the presence (heterozygous or polymorphic homozygous) of at least one polymorphic allele for each locus.

### Statistical analysis

The diagnoses and the corresponding follow-up periods for the retrospective cohort were reviewed independently by two hematologists, according to the new ELN recommendations [26]. That approach gave the possibility to gather together clinical records from both retrospective and prospective cohort of patients, without increasing the risk of potential biases.

For the main clinical variables, central indices (mean, median) and dispersion parameters (SD, range) were obtained. The analysis of variance (ANOVA) was used to evaluate every possible association between clinical efficacy measures and genetic variables. The Fisher's exact test was applied for the analysis of discrete variables, while the *t*-test was used for comparing the time to cytogenetic or molecular response and dichotomous variables.

The EFS was assessed from the date of initiation of treatment to the onset of any of the following events: absence of CCyR at 12 months, loss of CCyR and MR3 at any time during treatment, withdrawal of the drug for toxicity, progression to the accelerated/blast phase, any cause of death during the study. The probability of EFS was calculated using the Kaplan-Meier method.

Overall, the level of significance was set at p<0.05.

## Abbreviations

ABC: ATP binding cassette
AE: adverse event
CCyR: complete cytogenetic response
CHR: complete hematological response
CML: chronic myeloid leukemia
CTC-AE: Common Toxicity Criteria–Adverse Events
DMR: deep molecular response
ELN: European Leukemia Net
EFS: event-free survival
EMR: early molecular response
EUTOS: European Treatment and Outcome Study
hOCT1: human organic cation transport member 1
HWE: Hardy-Weinberg equilibrium
IS: international scale
MAF: minor allele frequency
MR3: major molecular response
SNP: single nucleotide polymorphism
TKI: tyrosine kinase inhibitor.
